# U.S. Women Faculty in the Social Sciences Also Face Gender Inequalities

**DOI:** 10.3389/fpsyg.2022.792756

**Published:** 2022-05-26

**Authors:** Bettina J. Casad, Christina E. Garasky, Taylor R. Jancetic, Anne K. Brown, Jillian E. Franks, Christopher R. Bach

**Affiliations:** ^1^Department of Psychological Sciences, University of Missouri–St. Louis, St. Louis, MO, United States; ^2^Department of Psychology, Brandeis University, Waltham, MA, United States; ^3^Department of Biology, Saint Louis University, St. Louis, MO, United States

**Keywords:** women faculty, gender bias, interventions—psychosocial/behavioral, social role theory, gender disparities in social sciences

## Abstract

There is a national interest in United States women’s underrepresentation in science, technology, engineering, and mathematics (STEM); however, gender inequality in the social sciences has not received similar attention. Although women increasingly earn postgraduate degrees in the social sciences, women faculty still experience gender inequities. Consistent gender inequities include slower career advancement, blunted salaries, unequal workloads, work-life conflict, systemic gender biases, underrepresentation in positions of power, and hostile work environments. Cultural biases suggest that once women have achieved parity, gender bias no longer exists. This review challenges that notion by providing evidence from social science domains in which women are well-represented but continue to face systemic gender biases. We examine cultural influences on gender representation and career advancement in psychology, economics, political science, sociology, and anthropology. We make interdisciplinary comparisons of career trajectories and salaries using national data, documenting patterns across the social sciences. For example, women economists face gendered standards in publishing, and women political scientists are less likely to have their work cited than men. Furthermore, data show that salaries become stagnant as the representation of women in these fields increases. These disparities reflect cultural biases in perceptions of women’s competence stemming from social role theory. We discuss best practices to address these problems, focusing on the ADVANCE organizational change programs funded by the National Science Foundation that target (a) improving academic climate, (b) providing professional development, and (c) fostering social networking. Federally supported interventions can reveal systemic gender biases in academia and reduce gender disparities for women academics in the social sciences.

## Introduction

There is a national interest in United States women’s underrepresentation and career advancement in the academic fields of science, technology, engineering, and mathematics (STEM); however, gender inequality in the social sciences has not received similar attention. While the number of women earning postgraduate degrees in the social sciences continues to increase, women faculty still experience gender inequities. Persistent gender inequities include slower careeradvancement, blunted salaries, unequal workloads, work–life conflict, systemic gender biases, underrepresentation in positions of power, and hostile work environments (Gruber et al., 2020). Cultural biases suggest that once women have achieved parity or are well-represented in an academic domain, gender bias no longer exists (Begeny et al., 2020). This review challenges that notion by providing evidence from social science domains in which women are well-represented but continue to face systemic gender biases (see Van Veelen and Derks, 2022). For example, women doctoral-level social scientists average $14,000 less than men regardless of academic rank (National Center for Science and Engineering Statistics [NCSES], 2021b).

This review examines United States cultural influences on gender representation and career advancement in psychology, economics, political science, sociology, and anthropology. For example, women economists face gendered standards in publishing, and women political scientists are less likely to have their work cited than their male peers (Maliniak et al., 2013). These disparities reflect cultural biases in perceptions of women’s competence stemming from social role theory (Eagly and Steffen, 1984). We make interdisciplinary comparisons of prestige and salaries using national data, documenting commonalities and differences across five social sciences.

We discuss best practices from effective interventions to address these problems, focusing on the ADVANCE organizational change programs funded by the National Science Foundation (NSF) that target (a) improving academic climate, (b) providing professional development, and (c) fostering social networking. Federally supported interventions implemented across the United States can reveal systemic gender biases in academia and enact solutions to reduce gender disparities for women academics in the social sciences.

## Social Role and Role Congruity Theoretical Frameworks

Even though women are typically well-represented among students and faculty in the social sciences, gender disparities persist (Gruber et al., 2020), which reflect long-standing cultural biases. Social role theory (Eagly and Steffen, 1984) is a helpful framework to understand the historical and continued gender disparities women face in academic careers. Historically, women did not have public education because their proper role in United States society was to be domestic caretakers. This role did not require formal education in the humanities and sciences (Welch and Ruelas, 2015). Later, when women obtained public education, they were limited to pursuing specific careers that fit feminine gender role expectations of caretaking (e.g., caring, nurturing), including secretaries, nurses, and early childhood education. According to role congruity theory (Eagly and Karau, 2002), women should fill normative social roles, including employment, compatible with the characteristics appropriate for women, such as prescriptive stereotypes to be warm, nurturing, and harmonious (Prentice and Carranza, 2002). Initially, women were not allowed to be schoolteachers. This intelligence domain was a masculine domain; only men had the competence and status to lead the future generation in intellectual pursuits. Over time, women became well represented among schoolteachers, and teaching became a primary profession for women due to the vital role of caring for young children and facilitating their intellectual and social development. During the “Republican Motherhood” (Kerber, 1976), women were put on a pedestal for their superior moral character and tasked with preparing the future generation of young republican boys to become workers, fathers, and heads of households.

Despite women’s dominance in youth education, the teaching profession was primarily a caretaking role rather than an intellectual pursuit. In contrast, men have historically dominated the professoriate. The highest levels of education were reserved for men as an advanced education was only necessary for individuals who were intellectually fit for such pursuits and those who engaged in paid employment and supported families.

The history of gendered social roles continues to influence women faculty’s experiences in academia. Although women have achieved significant advances, disparities persist, reflecting implicit biases. These implicit biases include perceptions of women as less competent than men, that women’s social roles should focus on nurturing, and that men should be awarded the appropriate status and prestige for their dominance in intellectual pursuits, e.g., in the form of salary and rank.

## Focus, Methodology, and Data Sources

Throughout this review, we provide evidence from five social science fields (psychology, economics, political science, sociology, anthropology) that cultural biases around gender role expectations may subtly maintain gender disparities in academia. These cultural biases affect women’s degree attainment, faculty ranks, salaries, time to tenure, leadership, authorship, publications, citations, conferences, networking, and grant funding.

### Focus

This review discusses trends and data from the United States. Although there is international interest in gender disparities in STEM and the social and behavioral sciences (e.g., the European Union’s Athena Swan Charter), the authors are most familiar with and work within the United States context. This review treats gender as a binary of cisgender women and men. National data sources (e.g., NSF) do not yet specify data for non-binary persons or other gender identities; therefore, our review reflects this cultural bias. Further, we acknowledge that women are not a monolithic group, and women’s experiences differ based on intersectional identities, including race, ethnicity, sexual orientation, age, and social class (Gruber et al., 2020). Women faculty from racially minoritized groups are less represented in academic domains than European American women and likely experience multiple disparities (Judson and Ross, 2021; Miles et al., 2021). Our review focuses on broad, cultural-level gendered patterns in academia, allowing for interdisciplinary comparisons. Our focus solely on gender is a limitation of the data. It reflects inadequacies in national datasets in delimiting data by subgroups (e.g., gender by race), which often have low sample sizes (Gruber et al., 2020). Our broad focus limits generalizability to women with intersectional identities (see Fox Tree and Vaid, 2022; Morimoto, 2022; Wong et al., 2022).

### Methodology

We selected the five social science disciplines of psychology, economics, political science, sociology, and anthropology due to their popularity (i.e., undergraduate enrollment) and greater representation among academic programs. These five fields are the most popular social science fields in terms of undergraduate degree attainment (Georgetown University, 2020), which often dictates the number of faculty in an academic department. However, most quantitative research studies on gender disparities in the social sciences focus on psychology, economics, and political science; thus, our examples come primarily from these fields. Much of the research on academic gender disparities in sociology and anthropology were case studies or qualitative, which we excluded. We exclude many other social sciences, but for comparative purposes, we provide data from the social sciences overall, which NSF defines (National Center for Science and Engineering Statistics [NCSES], 2017) to include the fields reviewed here and area and ethnic studies, history of science, linguistics, and others. By providing data from the social sciences as a broad category, we can evaluate how specific social science fields (e.g., sociology) compared to others, mainly whether patterns are similar or different as the representation of women varies across each discipline. Gender disparities exist in other fields, such as the humanities (e.g., philosophy); however, we excluded them to narrow the focus of the review.

### Data Sources

We used the most recent publicly available data sets that provide degree attainment by gender and field, median salaries, and representation within academic ranks. The data sources come from national government agencies (e.g., NSF) and professional disciplinary organizations (e.g., American Political Science Association [APSA], American Psychological Association [APA]). We also drew upon relevant scholarly literature reporting trends and patterns in academic gender disparities (e.g., Gruber et al., 2020; Casad et al., 2021).

## Women’s Representation, Academic Rank, and Salary in the Social Sciences

Women are well-represented in social science degree programs at all levels, accounting for 55.2% of baccalaureate, 57% of master’s, and 50.6% of doctoral degrees awarded (National Center for Science and Engineering Statistics [NCSES], 2019). We can compare representation within specific social science fields to the overall representation of women earning baccalaureate degrees in any field, which remains around 57% (National Center for Science and Engineering Statistics [NCSES], 2019). However, gender representation within specific social science fields varies, with women more highly represented in psychology, anthropology, and sociology but less represented in political science and economics (see Table 1). Research suggests gender representation within specific fields reflects how the domain promotes masculine cultural norms (Cheryan et al., 2017), consistent with a social role and role congruity framework.

**TABLE 1:** Gender representation in social science degree programs.

		Baccalaureate degrees awarded (%)	Master’s degrees awarded (%)	Doctoral degrees awarded (%)
Social Sciences	Men	44.8	43.0	49.4
	Women	55.2	57.0	50.6
Psychology	Men	21.1	19.8	26.3
	Women	78.9	80.2	73.7
Anthropology	Men	27.1	29.1	33.0
	Women	72.9	70.9	67.0
Sociology	Men	28.4	35.9	36.6
	Women	71.6	64.1	63.4
Political Science/Public Administration^a^	Men	45.1	43.1	52.0
	Women	54.9	56.9	48.0
Economics	Men	67.9	58.6	67.8
	Women	32.1	41.4	32.2

*Fields are listed from general social sciences to specific fields and most to least representation of women. Sources: National Center for Science and Engineering Statistics [NCSES] (2019), Argyle and Mendelberg (2020).*

*^a^NSF combines these subfields.*

### Gendered Patterns

In psychology, women outnumber men in degrees awarded at all levels (National Center for Science and Engineering Statistics [NCSES], 2019). However, these statistics obscure women’s lower representation in subfields of psychology, including cognitive neuroscience, cognitive psychology, experimental psychology, and neuropsychology (Hilsabeck and Martin, 2010; Vaid and Geraci, 2016; Odic and Wojcik, 2020; Fulvio et al., 2021). For example, compared to earning 73.7% of doctorates in psychology overall, women were awarded 53% of doctorates in cognitive neuroscience, 58.5% of doctorates in cognitive psychology and psycholinguistics, and 59.9% of doctorates in experimental psychology (National Center for Science and Engineering Statistics [NCSES], 2019). These statistics compare to women’s higher representation in other subfields, including 84.8% of doctorates in school psychology, 81.5% of doctorates in behavioral analysis, 86.4% of doctorates in development and child psychology, and 75.6% of doctorates in clinical psychology (National Center for Science and Engineering Statistics [NCSES], 2019).

Similar patterns to psychology emerge for women’s degree attainment in anthropology and sociology, which are higher than the social sciences overall but lower than their related social science discipline, psychology. In addition, women’s representation declines at the master’s and doctoral levels in anthropology and sociology.

Different patterns emerge in economics and political science, where women have lower overall representation than in psychology, anthropology, and sociology. Within economics, women’s representation is imbalanced across specialty areas. For example, women are scarce in general economics and finance and more abundant in labor and other applied microeconomic fields (Lundberg and Stearns, 2019). Although women trend toward equal or greater representation than men in political science at the baccalaureate (54.9%) and master’s (56.9%) levels, there is a lower representation at the doctoral level (48%); however, the inclusion of Public Administration in the National Center for Science and Engineering Statistics data may obscure gender representation.

### Theoretical Applications

Variation in women’s representation across education ranks and within specialty areas of psychology, economics, political science, anthropology, and sociology reflect gender role socialization. Women may gravitate toward subfields like developmental, school, and clinical psychology that meet communal goal affordances (Diekman et al., 2010), e.g., concerns for others’ welfare. Fields perceived as more agentic and prestigious have a lower representation of women, such as cognitive neuroscience, experimental psychology, sports sociology, and biological anthropology (Antón et al., 2018). Careers in anthropology and sociology, like psychology, involve the study of people, cultures, and societies and thus likely fill many women’s communal career goals (Diekman et al., 2010). People perceive economics as a profession that meets agentic career goals (Diekman et al., 2010). Given its focus on mathematics, many women may shy away from economics due to math-related stereotype threats, gender role socialization, and low math self-efficacy (Ceci et al., 2014; Cheryan et al., 2017). For political science, lower representation at the doctoral level is related to gender role socialization and stereotypes that politics and governmental power fall within the masculine domain (Mo, 2015).

## Faculty Rank and the Path to Tenure

Although women’s representation among degree earners in the social sciences has increased, their education does not directly translate into representation among the faculty ranks.

### Gendered Patterns

Except for economics and political science, there are more women in the lower faculty ranks than men, including Instructor/Lecturer and Assistant Professors, and less represented among Associate and Full Professors (see Table 2; Finder, 2007; Dellinger et al., 2009; Jaschik, 2009; American Political Science Association [APSA], 2011; Ginsberg, 2016; National Center for Science and Engineering Statistics [NCSES], 2017). Women hold most untenured instructor and lecturer positions in psychology, anthropology, and sociology (Finder, 2007; Jaschik, 2009; National Center for Science and Engineering Statistics [NCSES], 2017). Women’s representation in economics has stalled, with little to no progress in the past several decades, reflecting the lowest representation of women faculty, alongside physics, math, engineering, and far below biology and many other social science fields (Lundberg and Stearns, 2019).

**TABLE 2:** Gender representation among social science faculty positions by rank.

Social sciences	Instructor or lecturer (%)	Assistant professor (%)	Associate professor (%)	Full professor (%)
Men	49.5	54.5	54.6	70.6
Women	50.5	45.5	45.4	29.4
**Psychology**				
Men	31.1	34.2	44.2	54.0
Women	68.9	65.8	55.8	45.5
**Anthropology^a^**				
Men	33	51^a^	66	79
Women	67	49^a^	34	21
**Sociology, demography, and population studies^b^**				
Men	42.9	41.9	36.5	57.3
Women	57.1	58.1	63.5	42.7
**Political science and government^b^**				
Men	50.0	64.9	59.1	78.7
Women	50.0	35.1	40.9	21.3
**Economics**				
Men	66.7	61.4	71.0	84.9
Women	33.3	38.6	29.0	15.1

*Fields are listed from general social sciences to specific fields and most to least representation of women. All anthropology data come from Ginsberg (2016), Winking et al. (2019), and Burton et al. (2020), except for data on Assistant Professors, which reflects NSF data on Other Social Sciences; National Center for Science and Engineering Statistics [NCSES] (2017).*

*^a^NSF does not separately classify anthropology but includes it in Other social sciences.*

*^b^NSF combines these subfields.*

Even though women are numerically well-represented in the social sciences, gender disparities in time to tenure persist. In sociology, women are 29% less likely to achieve tenure than men and take longer to do so (men 6.6 years, women 7.2 years; Weisshaar, 2017). In a national study of 95 sociology departments assessing 475 randomly selected assistant professors in sociology, 78% of women received tenure compared to 85% of men (Weisshaar, 2017). After controlling for research productivity (e.g., publications and NSF grants), departmental characteristics, time in rank, and contextual factors, 40–45% of the variance in promotion and tenure remained unexplained, reflecting a gender bias in tenure evaluations (Weisshaar, 2017). In economics, 68% of men earn tenure within 10 years of earning their Ph.D. compared to 47% of women (Lundberg and Stearns, 2019). Women also take longer to earn tenure in political science and are less likely than men to be tenured at a research institution 10 years after earning their Ph.D. (American Political Science Association [APSA], 2004; Hesli et al., 2012).

The more male-dominated social sciences, specifically economics and political science, have attrition starting at the tenure stage. Although women make up 32% of doctoral recipients in economics (National Center for Science and Engineering Statistics [NCSES], 2020), women comprise only 15% of Full professors (National Center for Science and Engineering Statistics [NCSES], 2017). In addition, academia has the lowest representation of women economists in senior positions (Women in Economics Initiative, 2020). Political science also shows a loss of women at higher ranks, despite equitable representation at the baccalaureate and master’s levels (De Brey et al., 2021).

### Reasons for Gender Gaps in Faculty Rank and Time to Tenure

Research offers several explanations for gender disparities in rank and time to tenure. First, women are less likely to be promoted in fields in which they are overrepresented (Ceci et al., 2014), such as psychology, sociology, and anthropology. Women’s overrepresentation, albeit in lower ranks, may be interpreted by senior faculty and administrators as gender parity, and they may not see a need for intervention (Begeny et al., 2020). Secondly, women may hold themselves to higher standards for promotion than men and, therefore, may not seek promotion or delay consideration for promotion (Gruber et al., 2020). Previous research supports the tendency for women to hold themselves to higher standards, such as research on the shifting standards model (Biernat et al., 1997) or undervaluing their worth in pay allocations (O’Brien et al., 2012). It may also be that men overvalue their worth (Niederle et al., 2013; Niederle and Vesterlund, 2007; O’Brien et al., 2012). Finally, research on gender differences in competitiveness and risk aversion found that women were less likely to apply for a competitive tournament. However, when women did enter the competition, they were equally successful as men in a math-based challenge (Niederle et al., 2013; Niederle and Vesterlund, 2007).

Although research indicates that qualified women and men are equally likely to be hired in psychology tenure track positions (American Psychological Association [APA], 2017), this is not the case in economics (Steinpreis et al., 1999). Women economists report facing barriers that negatively affect their productivity and probability of promotion, which can reduce expectations of future success and impede research activity and publication outcomes (Lundberg and Stearns, 2019). Both popular media and scholarly sources note that economics is perceived to have a “dismal” climate for women, with “rampant” overt sexism and sexual harassment (Smith, 2014; Casselman and Tankersley, 2019; Wu, 2020). Further, letters of recommendation supporting job candidates’ applications for academic positions report different adjectives (e.g., agentic, communal) to describe men and women. The characteristics used to describe women are viewed more negatively in hiring decisions (Schmader et al., 2007; Madera et al., 2009). However, recent research indicates that letters for women faculty in psychology and sociology do not reflect gender differences compared to letters in physics and that letters in these social sciences favor women (Bernstein et al., 2022).

Another contribution to gender disparities in rank and time to tenure is the differential impact of parental leave. Women are more likely to take parental leave than men (Zagorsky, 2017), and research indicates that men’s productivity can benefit from parental leave (Antecol et al., 2018). Women in psychology without children and a partner are 8.7% more likely to receive tenure 6 years after earning their Ph.D. than men without children and a partner, providing evidence for the motherhood penalty (American Psychological Association [APA], 2017). Women faculty working toward tenure while having family responsibilities must contend with institutional policies that may hinder progress to tenure and promotion, such as flexibility regarding parental leave, stopping the tenure clock, and family-care reimbursement (Ginther, 2004). Such policies contribute to an academic climate in which women perceive they are devalued compared to men (Ginther, 2004).

### Theoretical Applications

The gender disparities in faculty rank and time to tenure reflect gender role socialization and implicit biases consistent with role congruity theory (Eagly and Karau, 2002). Women may be less represented in positions of power, that is, tenured Associate and Full Professor positions, due to implicit biases in hiring and promotion practices (Moss-Racusin et al., 2012; Devine et al., 2017; Gruber et al., 2020). Stereotypes of women as less competent than men persist and may leak into candidates’ letters of recommendation (Schmader et al., 2007; Madera et al., 2009), thus further biasing hiring and promotion. Women with children and partners seem to pay a “motherhood penalty” compared to men (Ginther, 2004; American Psychological Association [APA], 2017). Women are expected to fulfill communal roles, such as motherhood, whereas men are expected to be career oriented. Prescriptive gender stereotypes still influence the judgments of career women (Ginther, 2004; Rudman et al., 2012). Finally, gender role socialization and self-stereotyping play a role in women’s differential standards and perceptions of pay entitlement (Biernat et al., 1997; Laurin et al., 2011; Niederle et al., 2013; Niederle and Vesterlund, 2007; O’Brien et al., 2012), which reflect socialization to gender congruent roles.

## Financial Compensation

Data show salaries become stagnant as the representation of women in the social sciences increases, and career prestige similarly declines (e.g., American Psychological Association [APA], 2017). Gender gaps in salary remain despite equal rank, education, and experience, even in women-dominated social science fields (see Table 3). Despite equitable gender representation in degrees awarded in the social sciences, there are gender disparities in median annual salary across all types of employment. In 2019, women with a doctorate in any social science field earned a median annual salary of $92,000 compared to $110,000 for men (National Center for Science and Engineering Statistics [NCSES], 2021b). The gender gap in salaries across industries in the social sciences extends to academia. In all faculty ranks except for Instructors and Lecturers, men in the social sciences earn higher salaries than women, with the most significant gap (14k) at the Full Professor rank (National Center for Science and Engineering Statistics [NCSES], 2021a).

**TABLE 3:** Social science faculty salaries by gender and rank.

Social sciences	Instructor or lecturer	Assistant professor	Associate professor	Full professor
Men	59,000	80,000	90,000	129,000
Women	62,000	77,000	87,000	115,000
**Psychology**				
Men	60,000	77,000	89,000^b^	129,000
Women	65,000	75,000	89,000^b^	119,000
**Anthropology**				
Men	58,000	70,000	86,000	115,000
Women	61,000	73,000	84,000	107,000
**Sociology, demography, and population studies^a^**				
Men	52,000	75,000	83,000	129,000
Women	55,000	74,000	82,000	121,000
**Political science and government^a^**				
Men	64,000	74,000	82,000	122,000
Women	71,000	76,000	87,000	114,000
**Economics**				
Men	85,000	109,000^b^	109,000^b^	152,000
Women	76,000	96,000	103,000	129,000

*Fields are listed from general social sciences to specific fields and most to least representation of women. 2019 median salary (National Center for Science and Engineering Statistics [NCSES], 2021b). We do not assume men in Economics promoted from Assistant to Associate do not receive a raise. The values reflect the median rather than the mean and have different standard errors.*

*^a^NSF combines these subfields.*

*^b^Although the medians are equal, the standard errors differ (Psychology men 2k, women 2.5k; Economics Assistant 8k, Associate 5k).*

### Gendered Patterns

With the highest number of women faculty, disciplines such as psychology also have the greatest gender pay gaps (American Psychological Association [APA], 2017). Salary data from 2019 indicate that the median salary for men in psychology ($100,000) was higher than for women in psychology ($88,000; American Psychological Association [APA], 2019). Men earn more than women at all tenured/tenure-track psychology faculty ranks, with the greatest gap (10k) at the Full Professor level (National Center for Science and Engineering Statistics [NCSES], 2021b). In anthropology, women earn more than men at the lower ranks of Instructor/Lecturer ($61,000 vs. $58,000) and Assistant Professor ($73,000 vs. $70,000). However, at the higher ranks of Associate ($84,000 vs. $86,000) and Full ($107,000 vs. $115,000), men outearn women, with the greatest gap at the Full rank (8k; National Center for Science and Engineering Statistics [NCSES], 2021b). In sociology, women earn more than men ($55,000 to $52,000) only at the Instructor/Lecturer rank, and the gap widens in favor of men at the tenured/tenure-track ranks, with the greatest gap (8k) at the highest rank (National Center for Science and Engineering Statistics [NCSES], 2021b). In contrast to economics, women in political science have a lower salary gap, and at all but the Full Professor rank, outearn men (National Center for Science and Engineering Statistics [NCSES], 2021b; see Table 3). Regardless of academic rank, men earned more than women in economics, earning a median base annual salary of $123,000, whereas women earned $104,000 (National Center for Science and Engineering Statistics [NCSES], 2021b). In all fields except economics, women make more than men in the non-tenure-track ranks of Instructor or Lecturer.

### Theoretical Applications

Salaries become stagnant as the representation of women in the social sciences increases, and career prestige similarly declines (e.g., American Psychological Association [APA], 2017). More specifically, women’s salaries languish, but men’s do not, creating gender gaps in salary despite equal rank, education, and experience. Except for economics, the only rank in which women consistently earn more is the non-tenured instructor or lecturer positions. As a result, women are overrepresented in positions that provide the least power within the university. Significant systemic gender biases contribute to these disparities, such as devaluing women’s work (Ginther, 2004) and assuming men are the primary breadwinners in the home and therefore need higher salaries (Eagly and Karau, 2002). There also may be influences of perceived competence on salary related to scientific fields relying heavily on math and data analytic skills (e.g., economics). Stereotypes of women’s inferior abilities in math and science domains linger, and assumptions of men’s natural quantitative abilities may contribute to unequal pay in science domains (Ceci and Williams, 2007).

In addition to ongoing systemic bias, issues at the individual level persist due to gender role socialization. Women may undervalue their worth (O’Brien et al., 2012), whereas men may overvalue their worth (Niederle et al., 2013; Niederle and Vesterlund, 2007; O’Brien et al., 2012). This self-assessment bias (Correll, 2004) permeates the negotiating process. According to some research, women “just don’t ask” (Babcock and Laschever, 2003; Amanatullah and Morris, 2010). However, other research shows no gender differences in hiring salary negotiation practices (Crothers et al., 2010). Research shows differences in opening negotiations for promotion and related salary increases, with men initiating more than women, but these differences are slight and are moderated by situational ambiguity (Kugler et al., 2018).

Regardless of the causes of gender gaps in salaries, the gaps need attention to make progress toward gender equality. Legislation such as the Equal Pay Act (United States Department of Labor, 2021) can federally mandate equal pay for equal work in academia.

## Barriers to Women’s Representation and Career Success in the Social Sciences

Thus far, this manuscript has reviewed gender dominance, equity, and disparities in degree attainment, faculty ranks and the path to tenure, and salary in the social sciences. In addition to these critical areas of the education and academic pipelines, other barriers exist in various forms and stages of academic careers that hinder women’s career progression. Next, we address gender disparities in women’s academic experiences in leadership, authorship, publications, citations, social networks, and grant funding.

## Leadership

Within academia, women are underrepresented in leadership positions in the social sciences (Ceci et al., 2014; Gruber et al., 2020), such as Department Chair, Dean, Provost, President, and Chancellor. Women also are underrepresented in professional organization memberships (Gruber et al., 2020) and prestigious influential positions that guide the direction of the social science fields, such as journal editors and elected leaders in professional societies (Goodwin, 2005; Vaid and Geraci, 2016; American Psychological Association [APA], 2017).

### Gendered Patterns

Women are underrepresented in leadership positions in psychology departments and other areas of academic administration (American Psychological Association [APA], 2017). Despite outnumbering men in APA membership, women hold only 18% of APA editorships (American Psychological Association [APA], 2017). In 2013, the number of women editors in psychology journals dropped by 18%, putting the numbers on par with the number of women editors in 1995 (American Psychological Association [APA], 2017). Women in editorial positions in cognitive psychology and cognitive neuroscience subfields have seen less drop because there was never a rise. An analysis of ten leading journals that primarily focus on publishing topics from cognitive psychology indicates that 100% of the editors in chief were men, and men represented over 50% of the other editorial positions (Vaid and Geraci, 2016). After expanding the number of journals examined to include 60 cognitive psychology journals, researchers found that 80% of the editors and 70% of the associate editors were men (Vaid and Geraci, 2016). Women also are underrepresented as members and in leadership positions in some of the experimental and cognitive societies. For example, women made up about 15% of the Society of Experimental Psychology (Goodwin, 2005).

Women economics faculty report facing many challenges in pursuing tenure track positions. Combined with family responsibilities, they often are discouraged from pursuing leadership roles, particularly since they are already underrepresented among the tenure-track faculty (Ginther, 2004). Additionally, the university climate may discourage women in economics from pursuing more prestigious roles as women faculty report feeling devalued and experiencing sexism in the workplace (Ginther, 2004). Furthermore, women in economics receive less recognition and awards than men (Lundberg and Stearns, 2019), which may negatively impact their evaluation for leadership positions.

### Theoretical Applications

Research on leadership reports that women’s experience with the double bind of family responsibilities and working toward tenure and promotion creates hardships and perpetuates stereotypes (American Political Science Association [APSA], 2004). Consistent with role congruity theory, traits associated with leaders are not associated with motherhood (Hoyt and Simon, 2017). Additionally, systemic biases such as gender norms and stereotypes can put ambitious women in a double bind (Dittmar, 2015). Gender stereotypes regarding the traits necessary for leadership may put women, particularly mothers, at a disadvantage for prestigious leadership positions in political science and social sciences (Prentice and Carranza, 2002; Brescoll et al., 2018). Additionally, while outright hostility has decreased over time, researchers have found more resistance toward women Presidents (Streb et al., 2008), representing a considerable stigma associated with women in leadership roles.

Several studies show that when women express gender-specific stereotypes, it can reduce their support in leadership positions (Bauer, 2015; Mo, 2015). Furthermore, research indicates that women need to be more qualified to succeed in politics, whereas men often are accepted on potential (Mo, 2015). This disparity indicates that women are held to a higher standard than their male counterparts.

## Publications, Authorship, and Citations

A critical part of earning tenure and promotion is publishing and being cited by other researchers (Ghiasi et al., 2016; Mershon and Walsh, 2016). Unfortunately, women authors are underrepresented in top-tier journals within the social sciences (Gruber et al., 2020).

### Gendered Patterns

Although most academic sociologists are women, authorship does not reflect the representation of women (Weisshaar, 2017). For example, the number of women authors in the top sociology journals (*American Journal of Sociology*, *American Sociological Review*, and *Social Forces*) is disproportionately smaller compared to the number of men authors, as is the total number of women’s publications overall (Weisshaar, 2017; Lynn et al., 2019). In addition, women in the most prestigious sociology journals are less likely to be co-authors than men (Grant and Ward, 1991; Belgacem and Lamari, 2012).

Authorship positions reflect similar gender disparities. Senior, or last authorship, shows significant gender disparities, with women constituting 53.56% of last authors in developmental, 40.54% in clinical, and 34.48% in cognitive psychology (National Science Foundation [NSF], 2017). However, Odic and Wojcik (2020) found that the rates of women last authors in developmental, health, and clinical psychology have shown steady improvement. Women in political science are disproportionately less likely to be included in teams of co-authors (Teele and Thelen, 2017) and to be invited to contribute to edited volumes (Mathews and Andersen, 2001).

Gender disparities also exist in citation rates. Men authors are more likely to be cited than women authors in psychology (Gruber et al., 2020), economics (Maliniak et al., 2013), political science (Maliniak et al., 2013; Mitchell and Hesli, 2013; Mershon and Walsh, 2016; Dion et al., 2018), sociology (Weisshaar, 2017), and anthropology (Chibnik, 2014). In economics, women are less likely to cite themselves than men, and men tend to cite other men more than women (Maliniak et al., 2013). Gendered patterns in citations among the social sciences indicate papers authored by men as the first and last authors have been overcited compared with what would be expected based on the number of papers authored by male/male teams (Sarsons, 2015). Papers authored by teams with at least one woman in the first or last-author position have been under-cited, and in co-authored papers, men authors often are attributed more credit than women authors (Sarsons, 2015). Fulvio et al. (2021) note that the citation imbalance results from systemic factors.

### Theoretical Applications

The gender disparities in publishing, authorship position and citation patterns reflect implicit biases and differential standards based on gender stereotypes, reflected in predictions from role congruity theory (Eagly and Karau, 2002). Stereotypes of women as less competent than men permeate judgments of women’s scholarship, as reflected in evaluation standards. Both men and women reviewers hold women authors to a higher standard (as measured by citation counts; Lundberg and Stearns, 2019). Additionally, men’s and women’s publications are evaluated differently, such that women with more co-authored publications are less likely to receive tenure than similar men (Sarsons, 2015). Women may face higher expectations because of gender gaps in publication rates and thus feel the need to work more to keep up with their men colleagues (Correll et al., 2017).

Men and women alike hold implicit biases about gender that shape their attitudes and behavior including the tendency to think of—and reference—men rather than women as experts (Morrow-Jones and Box-Steffensmeier, 2014; Leslie et al., 2015). When deadlines are looming, academics often reach for the most accessible and known literature, usually authored by men (Beaulieu et al., 2017). The citation bias favoring men in political science and methodologically focused social sciences is so familiar that it is called the “Matthew Effect” (Dion et al., 2018). The bias against women, the “Matilda Effect,” excludes women’s research citations from articles, scholarly journals, course syllabi, and textbooks (Dion et al., 2018). Publication and citation biases negatively impact academics careers, considering the significant impact citations and exposure have on consideration for raises, tenure and promotion, grants, and research awards.

Regarding potential self-stereotyping and differential standards, research in sociology suggests that women only submit their best writing compared to men authors, who are more likely to submit a broader range of quality of writing (Reuben et al., 2014). With this line of reasoning, one would expect women’s publications to be higher quality and, thus, more likely to get published than male-led papers, though research shows otherwise (Lynn et al., 2019). This discrepancy in evaluation can lead to substantial differences in the probability that women-authored papers receive a revise and resubmit decision.

While the evidence is not conclusive, differences in co-authorship networks and potential bias in the publishing process may contribute to this gap. The Committee on the Status of Women in the Economics Profession Mentoring Program (CeMent) significantly increased the publication rates of participants by 20%, bolstering the argument that lack of mentoring may be a significant contribution to women’s lower authorship (Blau et al., 2010). In anthropology, women are more likely to get published in journals with at least one woman editor (McElhinny et al., 2003).

## Social Networks and Conference Presentations

Interventions promoting women in academia often focus on facilitating the development of their social networks (Casad et al., 2021). Women need robust social networks because of gender gaps in publication rates, authorship positions, and citations. However, each social science reviewed here indicates that insufficient social networks play a role in women’s lower representation in higher faculty ranks, leadership positions, publications, authorship, and citation rates (American Political Science Association [APSA], 2004; Lundberg and Stearns, 2019). One way to increase recognition and reputation and increase one’s scholarly network is to present research at conferences (Carley and Wendt, 1991). Next, we describe gender disparities in conference presentations and issues with social networks.

### Gendered Patterns

Women are underrepresented at high-profile conferences in psychology (Hinshaw et al., 2014; Johnson et al., 2017), economics (Lundberg and Stearns, 2019), and anthropology (Isbell et al., 2012), more likely to present at regional than international conferences (Hinshaw et al., 2014), and more likely to present posters than talks (Hinshaw et al., 2014). For example, from 2013 to 2016, the National Bureau of Economic Research Summer Institute Conference had only 20.6% women authors (Chari and Goldsmith-Pinkham, 2017). Similarly, women in political science are disproportionately less likely to appear on professional panels at conferences and be invited to speak at university colloquia (Nittrouer et al., 2018). An examination of sociology colloquia speakers at the top 50 colleges and universities in the United States indicated that men were more likely to be invited speakers than women. This gender disparity was not explained by women declining invitations or viewing colloquium talks as unimportant (Nittrouer et al., 2018). This pattern also existed for psychology and political science colloquia (Nittrouer et al., 2018). Research also suggests that political science conferences encourage a masculine normative culture (Biggs et al., 2018). When women are missing from academic discussions, the professions lose out on the expertise and perspective they have to offer (Barnes and Beaulieu, 2017), and faculty miss exposure, networking, and potential job opportunities (Boss and Eckert, 2004; Nittrouer et al., 2018).

In addition to representation as speakers at conferences, women experience disparate treatment at professional meetings than men. Women presenters often are asked 3–6 more questions on average than men presenters (Dupas et al., 2021). Men were more likely to ask questions and offer comments to women than men presenters, suggesting higher rates of critical feedback for women, resulting in the audience’s adverse reaction (Winking et al., 2019). A higher rate of questioning, particularly by men in the audience, may reflect perceptions of women’s lower competence and may create more hostile environments for women at conferences.

In contrast to potentially hostile environments at psychology, economics, political science, and anthropology conferences, after accounting for speaker and audience gender composition, women at sociology conferences tend to have equal speaking time as men (Kriwy et al., 2013). However, when the audience was primarily women, women tended to have more speaking time, mainly when women Associate and Full Professors were in the majority (Kriwy et al., 2013). This finding suggests that women-dominant networks are beneficial to women as they provide gender capital and gender equity to women in the professional career domain (McAdam et al., 2019).

### Theoretical Applications

Conferences and social networks are yet additional intellectual domains in which women are underrepresented. The exact causes of these gender disparities are unknown, but they likely reflect gendered socialization in professional development and professional cultural norms. For example, speaking at a conference is prestigious and reflects one’s prominence in their field. If women are underrepresented, receive more critical feedback, and have less access to social networks than men, they are further disadvantaged in intellectual domains, consistent with a role congruity perspective of academic gender disparities.

## Grant Funding

The gender gap in success rates for research funding is prevalent in the social sciences (i.e., psychology and anthropology; Van der Lee and Ellemers, 2015). Research indicates gender equality at the application stage of funding, but disparities emerge at the award level (Van der Lee and Ellemers, 2015). For example, some research indicates slight gender bias in the funding of National Institutes of Health (NIH) R01 grants (Forscher et al., 2019), yet other research shows women earn smaller grant awards, nearly $40,000 less (Oliveira et al., 2019). In addition, Biernat et al. (2020) suggest that women may respond more negatively to feedback and be less likely to resubmit a grant than men (Biernat et al., 2020). Finally, research shows bias in the narratives of grant peer reviews (Magua et al., 2017).

### Gendered Patterns

According to the American Psychological Association [APA], 2017, women tenure-track faculty are less likely to receive research grants. The NIH reports that women received 35% of the Research Project Grants, such as an R01 grant, in the 2020 fiscal year (Chaudhary et al., 2021). NIH grant awards indicate no gender differences in the number of Principal Investigators awarded a first-time grant; however, only 31% of NIH grantees are women (Hechtman et al., 2018). When women earn NIH grants, they are less likely to apply for renewals or other grants later in their careers (Boyle et al., 2015; Hechtman et al., 2018). Overall, women are less likely to apply for research grants but have an equal likelihood of funding as men when reviewers focus on the quality of the proposed research rather than the investigator’s credentials (Gruber et al., 2020).

Many social scientists seek funding from the NSF rather than, or in addition to, the NIH. Research indicates that women are less likely to submit grants as Principal Investigators to the Directorate of Social, Behavioral, and Economic Sciences than men, even after considering their representation in academia (Rissler et al., 2020). Men’s NSF submission rate is a 1:1 ratio of submissions to male faculty in academia (Rissler et al., 2020). Although gender differences in NSF grant submissions exist, data suggest equal funding success rates (Rissler et al., 2020). Next, we turn to theoretical applications for these gendered patterns in grant applications and funding.

### Theoretical Applications

Researchers’ interpretation of gender gaps in funding takes (Eagly, 2020) or complements a social role theory lens (Rissler et al., 2020). Women tend to work in more teaching-intensive than research-intensive colleges and universities, which put less emphasis on research for tenure and promotion (Eagly, 2020). In teaching-intensive roles, there is less incentive to submit NIH or NSF research grants (Rissler et al., 2020). Similarly, women are less likely to indicate that research is their primary responsibility (Rissler et al., 2020), even at very high research universities. Instead, women more often engage in teaching, mentoring, service, and other non-research-related responsibilities (e.g., administration; Mitchell and Hesli, 2013; O’Meara et al., 2017), which take away time and focus from grant submissions. Roles in which women dominate, such as teaching, mentoring, and service, are perceived to be more communal. In contrast, research is more agentic, which may influence women’s focus in academic careers if they are communal goal oriented.

## Lessons From Interventions

Federal granting agencies like the NSF and NIH earmark funding to address gender disparities in STEM; however, fewer funding mechanisms target gender equity in the social sciences. Despite the primary focus on STEM, several NSF and NIH-funded interventions include faculty from the social and behavioral sciences. NSF’s ADVANCE program expanded STEM to include social science fields (i.e., psychology, economics, sociology, and political science; Hutchins and Kovach, 2019). We review the main findings of effective interventions funded by the NSF, NIH, universities, and private foundation grants to demonstrate minor changes that can combat inequality in STEM. The social sciences can reduce the gender disparities addressed in this review. Successful interventions addressing inequality within the social sciences often focus on (a) improving academic climate, (b) providing professional development, and (c) fostering social networking.

As stated throughout this review, stereotypes and biases in the social sciences lead to workload inequities and hostile academic climates for women (Moss-Racusin et al., 2012; Devine et al., 2017; Gruber et al., 2020). Interventions to improve academic climate include the Faculty Workload and Rewards Project (FWRP: The Faculty Workload and Rewards Project [umd.edu]), Athena Swan Charter (Athena Swan Charter | Advance HE [advance-he.ac.uk], the Recruitment of Underrepresented People (GEAR UP; GEAR UP: Faculty Search Committee Training Program | University of New Hampshire [unh.edu]), and Transformation through Relatedness, Autonomy, and Competence Support program (TRACS; https://www.montana.edu/nsfadvance/summary.html), which have been successful at addressing gender workload inequalities (e.g., campus service, teaching, and mentoring workloads) and workload equity reform (e.g., providing resources, giving credit where credit is due, challenging *status quo* thinking and distrust). In addition, understanding how implicit bias impacts faculty workload empowers women to seek additional departmental support. Removing implicit bias in workload can be addressed by focusing on workload transparency (e.g., faculty workload activity dashboards, faculty service audits), clarity (e.g., faculty expectation guidelines, compensation for crucial roles), and credit (e.g., credit systems, teaching credit swaps). In addition, norms (e.g., planned service rotations, planned teaching-time rotations), context (e.g., differentiated workload policy, modified criteria for promotion and tenure), and accountability (e.g., restructuring and reducing committees, statement of mutual expectations) reduce bias (O’Meara et al., 2017, 2020). Educating faculty about microaggressions and biases and how to address them effectively changes departmental climate, improves workplace satisfaction, and increases perceptions of fairness and self-advocacy for all faculty involved (i.e., white men, women, racially minoritized groups; O’Meara et al., 2017, 2020).

Interventions that raise awareness of microaggressions and implicit biases directly influence hiring practices. Faculty reported that bias education increased their understanding of how gender impacts the evaluation of job candidates and how microaggressions and implicit biases impact candidate selection. This improved understanding leads to an increase of between 20 (Jones et al., 2019) and 67% (Smith et al., 2015) of women faculty representation. Successful workplace equity interventions demonstrate that educating faculty about workload inequalities and gender biases in academia and working together to implement changes positively influence women faculty and increase the representation of women in the social sciences.

In addition to fostering equitable climates, interventions such as the Visiting STEM Women Scholars Program (Visiting STEM Women Scholars Program [unh.edu]), the Gender Equity Project (Gender Equity Benchmarks — Hunter College [cuny.edu]), and TRACS (Smith et al., 2017) focus on advancing women’s achievement in academia, including the social sciences. These interventions include workshops informing women on enhancing research opportunities, improving grant proposals, building research labs, mentoring graduate students, and achieving work-life balance while providing opportunities for underrepresented faculty to increase recognition within their fields (Smith et al., 2015). For example, after participating in the TRACS grant-writing boot camps, women faculty submitted more external grants, served as principal investigators on more proposals, and received more external grant funds than their pre-workshop achievements and a comparison sample of non-TRACS peers (Smith et al., 2017). These interventions increase research funding and scholarly productivity and decrease attrition (Hunter College, 2007; Barnes and Beaulieu, 2017).

Another effective intervention for supporting women and increasing their social sciences representation is building social networks. Interventions like the Visions in Methodology group (VIM; VIM | Visions in Methodology) and the American Economic Association (AEA) Committee on the Status of Women in the Economics Profession’s (CSWEP) Mentoring Program (CeMENT; https://www.aeaweb.org/about-aea/committees/cswep/programs/cement-mentoring-workshops) introduce junior women faculty to senior women faculty. These partnerships allow faculty members to share knowledge on the tenure process, build peer networks (Blau et al., 2010), and understand career success factors (e.g., publishing, effective teaching, work–life balance). Social network interventions build faculty networks and increase women’s sense of support from their networks (e.g., mentors and peers) compared to women at similar points in their careers without such networks (Barnes and Beaulieu, 2017).

While this review examines interventions that address one or more of three topics, (a) improving academic climate, (b) providing professional development, and (c) fostering social networking, many successful interventions address multiple factors related to gender inequalities. For example, interventions to increase women’s achievements or decrease biases against women may also have a mentorship component. Additionally, interventions focusing on one aspect of gender inequality still led to change in other domains. Mentorship interventions increase support and recognition one has in one’s field, thus increasing achievement and strengthening social networks. For example, women who attended VIM conferences that focus on faculty mentorship and career support submitted significantly more articles per year on average (2.23) than comparable women who did not attend VIM conferences (1.58), which is like comparable men faculty (1.96; Barnes and Beaulieu, 2017). Furthermore, women who attended the VIM conferences gave 0.48 more talks than comparable men, on average, the following year, and 0.60 more talks during their careers than other women at similar points (Barnes and Beaulieu, 2017). On average, women who attended CeMENT workshops received 0.4 more NSF and NIH grants, were 25% more likely to have a top-tier publication, and had, on average, three additional publications than women in a comparison group five years after the intervention (Blau et al., 2010). These statistics demonstrate that interventions that (a) improve academic climate, (b) provide professional development, and (c) foster social networking can impact women social scientists’ success and address several factors that cause gender inequalities.

## Conclusion

This review and others (e.g., Gruber et al., 2020) provide evidence that examining gender disparities in the social sciences is warranted. Less national attention and federal funding have focused on gender inequities in the social sciences because many of these fields have better representation of women (American Psychological Association [APA], 2017; Begeny et al., 2020; Gruber et al., 2020; Van Veelen and Derks, 2022). Despite higher degree attainment among women in the social sciences, psychology, anthropology, and sociology, women faculty are underrepresented at higher faculty ranks and among economics and political science faculty. Several peer-reviewed studies document systemic biases women faculty face in hiring, promotion, tenure, salaries, leadership positions, authorship, publications, citations, conferences and social networking, and grant funding.

Social role and role congruity theories and the examination of communal and agentic goals and implicit biases provide an explanatory framework for persisting gender stereotypes and broader systemic gender biases in social science fields. In sum, evidence indicates that cultural gender biases subtly maintain gender disparities in academia in degree attainment, faculty ranks, salaries, time to tenure, leadership, authorship, publications, citations, conferences, networking, and grant funding.

Through the NSF ADVANCE program, federal research funding and other agencies (e.g., NIH) have targeted academic interventions to reduce gender disparities. Much of this work focuses on STEM, but many programs include the social and behavioral sciences in interventions and policy changes. This review highlights several successful interventions that focus on changing organizational cultures, policies, and practices that continue to disenfranchise women in academia. In addition, interventions provide training to improve academic climates, promote professional development, and foster social networking opportunities to enrich the professional lives of women in the social sciences.

## Author Contributions

BC contributed to conception and design of the study, wrote the introduction, abstract, and conclusion, and completed all editing. CG wrote the interventions section. TJ wrote on Anthropology and Sociology. AB wrote on Economics and Political Science. JF wrote on Psychology, provided statistics, and created tables. CB wrote on Social Sciences, provided statistics, and created tables. All authors contributed to manuscript revision, read, and approved the submitted version.

## Conflict of Interest

The authors declare that the research was conducted in the absence of any commercial or financial relationships that could be construed as a potential conflict of interest.

## Publisher’s Note

All claims expressed in this article are solely those of the authors and do not necessarily represent those of their affiliated organizations, or those of the publisher, the editors and the reviewers. Any product that may be evaluated in this article, or claim that may be made by its manufacturer, is not guaranteed or endorsed by the publisher.

## References

[B1] Amanatullah E. T. Morris M. W. (2010). Negotiating gender roles: gender differences in assertive negotiating are mediated by women’s fear of backlash and attenuated when negotiating on behalf of others. *J. Pers. Soc. Psychol.* 98 256–267. 10.1037/a0017094 20085399

[B2] American Political Science Association [APSA] (2004). *Women’s Advancement in Political Science: A Report of the APSA Workshop on the Advancement of Women in Academic Political Science in the United States.* Washington, DC: APSA.

[B3] American Political Science Association [APSA] (2011). *Report of the Task Force on Political Science in the 21st Century.* Washington, DC: APSA.

[B4] American Psychological Association [APA] (2017). *The Changing Gender Composition of Psychology: Update and Expansion of the 1995 Task Force Report.* Washington, DC: APA.

[B5] American Psychological Association [APA] (2019). *The Academic Psychology Workforce: Characteristics of Psychology Research Doctorates in Faculty Positions (1995-2015).* Washington, DC: APA.

[B6] Antecol H. Bedard K. Stearns J. (2018). Equal but inequitable: who benefits from gender-neutral tenure clock stopping policies? *Am. Econ. Rev.* 108 2420–2441. 10.1257/aer.20160613

[B7] Antón S. C. Malhi R. S. Fuentes A. (2018). Race and diversity in U.S. Biological Anthropology: a decade of AAPA initiatives. *Am. J. Phys. Anthropol.* 165 158–180. 10.1002/ajpa.23382 29380881

[B8] Argyle L. P. Mendelberg T. (2020). Improving women’s advancement in political science: what we know about what works. *Polit. Sci. Polit.* 53 718–722. 10.1017/s1049096520000402

[B9] Babcock L. Laschever S. (2003). *Women don’t ask: Negotiation and the gender divide.* Princeton, NJ: Princeton University Press.

[B10] Barnes T. D. Beaulieu E. (2017). Engaging women: addressing the gender gap in women’s networking and productivity. *Polit. Sci. Polit.* 50 461–466. 10.1017/s1049096516003000

[B11] Bauer N. M. (2015). Emotional, sensitive, and unfit for office: gender stereotype activation and support for female candidates. *Polit. Psychol.* 36 691–708. 10.1111/pops.12186

[B12] Beaulieu E. Boydstun A. E. Brown N. E. Dionne K. Y. Gillespie A. Klar S. (2017). Women also know stuff: meta-level mentoring to battle gender bias in political science. *Polit. Sci. Polit.* 50 779–783. 10.1017/s1049096517000580

[B13] Begeny C. T. Ryan M. K. Moss-Racusin C. A. Ravetz G. (2020). In some professions, women have become well represented, yet gender bias persists—Perpetuated by those who think it is not happening. *Sci. Adv.* 6:eaba7814. 10.1126/sciadv.aba7814 32637616 PMC7319752

[B14] Belgacem I. Lamari M. (2012). “Scientists’ collaboration in the social sciences field: investigating the determinants of scholarly collaboration in the Canadian context 2001–2008,” in *Proceedings of the International Conference on Education and e-Learning Innovations*, New York, NY.

[B15] Bernstein R. H. Macy M. W. Williams W. M. Cameron C. J. Williams-Ceci S. C. Ceci S. J. (2022). Assessing gender bias in particle physics and social science recommendations for academic jobs. *Soc. Sci.* 11:74. 10.3390/socsci11020074

[B16] Biernat M. Carnes M. Filut A. Kaatz A. (2020). Gender, race, and grant reviews: translating and responding to research feedback. *Pers. Soc. Psychol. Bull.* 46 140–154. 10.1177/0146167219845921 31088206 PMC7065520

[B17] Biernat M. Manis M. Kobrynowicz D. (1997). Simultaneous assimilation and contrast effects in judgments of self and others. *J. Pers. Soc. Psychol.* 73:254. 10.1037//0022-3514.73.2.254 9248048

[B18] Biggs J. Hawley P. H. Biernat M. (2018). The academic conference as a chilly climate for women: effects of gender representation on experiences of sexism, coping responses, and career intentions. *Sex Roles* 78 394–408. 10.1007/s11199-017-0800-9

[B19] Blau F. Currie J. Croson R. T. A. Ginther D. (2010). Can mentoring help female Assistant Professors? Interim results from a randomized trial. *Am. Econ. Rev.* 100 348–352. 10.3386/w15707

[B20] Boss J. M. Eckert S. H. (2004). *Academic Scientists at Work: The Job Talk. Science.* Available online at: www.sciencemag.org/careers/2004/12/academic-scientists-work-job-talk (accessed October 9, 2021).

[B21] Boyle P. Smith L. Cooper N. (2015). Gender balance: women are funded more fairly in social science. *Nature* 525 181–183. 10.1038/525181a 26354468

[B22] Brescoll V. L. Okimoto T. G. Vial A. C. (2018). You’ve come a long way…maybe: how moral emotions trigger backlash against women leaders. *J. Soc. Issues* 74 144–164. 10.1111/josi.12261

[B23] Burton M. Watson P. J. Webster C. (2020). *Academic Employment of Women: Participate & Advocate.* Available online at: https://www.americananthro.org/ParticipateAndAdvocate/Content.aspx?ItemNumber=12674 (accessed October 9, 2021).

[B24] Carley K. Wendt K. (1991). Electronic mail and scientific communication. A study of the SOAR extended research group. *Knowledge* 12 406–440. 10.1177/107554709101200405

[B25] Casad B. J. Franks J. E. Garasky C. E. Kittleman M. M. Roesler A. C. Hall D. Y. (2021). Gender inequality in academia: problems and solutions for women faculty in STEM. *J. Neurosci. Res.* 99 13–23. 10.1002/jnr.24631 33103281

[B26] Casselman B. Tankersley J. (2019). *Women in Economics Report Rampant Sexual Assault and Bias.* New York, NY: The New York Times.

[B27] Ceci S. J. Ginther D. K. Kahn S. Williams W. M. (2014). Women in academic science: a changing landscape. *Psychol. Sci. Public Interest* 15 75–141. 10.1177/1529100614541236 26172066

[B28] Ceci S. J. Williams W. M. (2007). *Why Aren’t More Women in Science?: Top Researchers Debate the Evidence.* Washington, DC: American Psychological Association.

[B29] Chari A. Goldsmith-Pinkham P. (2017). *Gender Representation in Economics Across Topics and Time: Evidence from the NBER Summer Institute (No. w23953).* Cambridge, MA: National Bureau of Economic Research.

[B30] Chaudhary A. Naveed S. Safdar B. Saboor S. Zeshan M. Khosa F. (2021). Gender differences in research project grants and R01 grants at the National Institutes of Health. *Cureus* 13:e14930. 10.7759/cureus.14930 34123629 PMC8188626

[B31] Cheryan S. Ziegler S. A. Montoya A. K. Jiang L. (2017). Why are some STEM fields more gender-balanced than others? *Psychol. Bull.* 143 1–35. 10.1037/bul0000052 27732018

[B32] Chibnik M. (2014). Gender and citations in american anthropologist. *Am. Anthropol.* 116 493–496. 10.1111/aman.12117

[B33] Correll S. J. (2004). Constraints into preferences: gender, status, and emerging career aspirations. *Am. Sociol. Rev.* 69 93–113. 10.1177/000312240406900106

[B34] Correll S. J. Ridgeway C. L. Zuckerman E. W. Jank S. Jordan-Bloch S. Nakagawa S. (2017). It’s the conventional thought that counts: how third-order inference produces status advantage. *Am. Sociol. Rev.* 82 297–327. 10.1177/0003122417691503

[B35] Crothers L. M. Hughes T. L. Schmitt A. J. Theodore L. A. Lipinski J. Bloomquist A. J. (2010). Has equity been achieved? Salary and promotion negotiation practices of a national sample of school psychology university faculty. *Psychol. Manag. J.* 13 40–59. 10.1080/10887150903553790

[B36] De Brey C. Snyder T. D. Zhang A. Dillow S. A. (2021). *Digest of Education Statistics 2019 (NCES 2021-009).* Washington, DC: National Center for Education Statistics, Institute of Education Sciences, U.S. Department of Education.

[B37] Dellinger K. England P. Nelson M. Robnett B. Vidal-Ortiz S. Spalter-Roth R. (2009). *Report of the American Sociological Association’s Committee on the Status of Women in Sociology.* Washington, DC: American Sociology Association.

[B38] Devine P. G. Forscher P. S. Cox W. T. Kaatz A. Sheridan J. Carnes M. (2017). A gender bias habit-breaking intervention led to increased hiring of female faculty in STEMM departments. *J. Exp. Soc. Psychol.* 73 211–215. 10.1016/j.jesp.2017.07.002 29249837 PMC5729935

[B39] Diekman A. B. Brown E. R. Johnston A. M. Clark E. K. (2010). Seeking congruity between goals and roles: a new look at why women opt out of science, technology, engineering, and mathematics careers. *Psychol. Sci.* 21 1051–1057. 10.1177/0956797610377342 20631322

[B40] Dion M. L. Sumner J. L. Mitchell S. M. L. (2018). Gendered citation patterns across political science and social science methodology fields. *Polit. Analysis* 26 312–327. 10.1017/pan.2018.12

[B41] Dittmar K. (2015). *Navigating Gendered Terrain: Stereotypes and Strategy in Statewide Races.* Philadelphia, PA: Temple University Press.

[B42] Dupas P. Modestino A. S. Niederle M. Wolfers J. (2021). *Gender and the Dynamics of Economics Seminars (No. w28494).* Cambridge, MA: National Bureau of Economic Research.

[B43] Eagly A. H. (2020). Do the social roles that women and men occupy in science allow equal access to publication? *Proc. Natl. Acad. Sci. U.S.A.* 117 5553–5555. 10.1073/pnas.2001684117 32127488 PMC7084134

[B44] Eagly A. H. Karau S. J. (2002). Role congruity theory of prejudice toward female leaders. *Psychol. Rev.* 109 573–598. 10.1037/0033-295X.109.3.573 12088246

[B45] Eagly A. H. Steffen V. J. (1984). Gender stereotypes stem from the distribution of women and men into social roles. *J. Pers. Soc. Psychol.* 46 735–754. 10.1037/0022-3514.46.4.735

[B46] Finder A. (2007). *Decline of The Tenure Track Raises Concerns.* New York, NY: New York Times.

[B47] Forscher P. S. Cox W. T. Brauer M. Devine P. G. (2019). Little race or gender bias in an experiment of initial review of NIH R01 grant proposals. *Nat. Hum. Behav.* 3 257–264. 10.1038/s41562-018-0517-y 30953009 PMC6699508

[B48] Fox Tree J. E. Vaid J. (2022). Why so few, still? Challenges to attracting, advancing, and keeping women faculty of color in academia. *Front. Sociobiol.* 6:792198. 10.3389/fsoc.2021.792198 PMC8804352 35118155

[B49] Fulvio J. M. Akinnola I. Postle B. R. (2021). Gender (im)balance in citation practices in cognitive neuroscience. *J. Cogn. Neurosci.* 33 3–7. 10.1162/jocn_a_01643 33078992 PMC9811856

[B50] Georgetown University (2020). *National Data for Social Sciences Majors.* Washington, DC: Georgetown University.

[B51] Ghiasi G. Larivière V. Sugimoto C. (2016). “Gender differences in synchronous and diachronous self-citations,” in *Proceedings of the 21ST International Conference on Science and Technology Indicators*, eds Ràfols I. Molas-Gallart J. Castro-Martínez E. Woolley R. (Valencia: Editorial Universitat PoliteÌcnica de ValeÌncia), 844–851.

[B52] Ginsberg D. (2016). *Survey Respondents At-A-Glance: 2016 Membership Survey, Report #1.* Arlington: American Anthropological Association.

[B53] Ginther D. K. (2004). *Gender Differences in Salary and Promotion in Political Science.* Available online at: https://citeseerx.ist.psu.edu/viewdoc/download?doi=10.1.1.595.4036&rep=rep1&type=pdf (accessed October 9, 2021).

[B54] Goodwin C. J. (2005). Reorganizing the experimentalists: the origins of the society of experimental psychology. *History Psychol.* 8 347–361. 10.1037/1093-4510.8.4.347 17152747

[B55] Grant L. Ward K. B. (1991). Gender and publishing in sociology. *Gender Soc.* 5 207–223. 10.1177/089124391005002005

[B56] Gruber J. Mendle J. Lindquist K. A. Schmader T. Clark L. A. Bliss-Moreau E. (2020). The future of women in psychological science. *Perspect. Psychol. Sci.* 16 483–516. 10.1177/1745691620952789 32901575 PMC8114333

[B57] Hechtman L. A. Moore N. P. Schulkey C. E. Miklos A. C. Calcagno A. M. Aragon R. (2018). NIH funding longevity by gender. *Proc. Natl. Acad. Sci. U.S.A.* 115 7943–7948. 10.1073/pnas.1800615115 30012615 PMC6077749

[B58] Hesli V. L. Lee J. M. Mitchell S. M. (2012). Predicting rank attainment in political science: what else besides publications affects promotion? *Polit. Sci. Polit.* 45 475–492. 10.1017/s1049096512000364

[B59] Hilsabeck R. C. Martin E. M. (2010). Women and advancement in neuropsychology: real-life lessons learned. *Clin. Neuropsychol.* 24 481–492. 10.1080/13854040802360566 18841516 PMC3624901

[B60] Hinshaw J. Reinbold T. Geher M. E. Geher G. (2014). Are women given voice at psychology conferences? A content analysis of gender of presenter at major evolutionist and general psychology conferences. *J. Evol. Stud. Consort.* 6 17–24.

[B61] Hoyt C. L. Simon S. (2017). “Social psychological approaches to women and leadership theory,” in *Handbook of Research on Gender and Leadership*, ed. Madsen S. R. (Cheltenharn: Edward Elgar Publishing), 85–99. 10.4337/9781785363863.00013

[B62] Hunter College (2007). *The Gender Equity Project: Leading the way in demolishing the glass ceiling–Benchmarks 2006-2007.* Available online at: http://www.hunter.cuny.edu/genderequity/ (accessed October 9, 2021).

[B63] Hutchins H. M. Kovach J. V. (2019). ADVANCING women academic faculty in STEM careers: the role of critical HRD in supporting diversity and inclusion. *Adv. Dev. Hum. Resour.* 21 72–91. 10.1177/1523422318814547

[B64] Isbell L. A. Young T. P. Harcourt A. H. (2012). Stag parties linger: continued gender bias in a female-rich scientific discipline. *PLoS One* 7:e0049682. 10.1371/journal.pone.0049682 PMC3504155 23185407

[B65] Jaschik S. (2009). *The Disappearing Tenure-Track Job.* Stanford, CA: Stanford University.

[B66] Johnson C. S. Smith P. K. Wang C. (2017). Sage on the stage: women’s representation at an academic conference. *Pers. Soc. Psychol. Bull.* 43 493–507. 10.1177/0146167216688213 28903659

[B67] Jones W. Graham K. Shea C. M. Williams J. (2019). *UNH UNBIASED: Leadership Development and Policy Change to Promote Institutional Transformation.* Available online at: https://ssrn.com/abstract=3345185 (accessed October 9, 2021).

[B68] Judson E. Ross L. (2021). Effects of gender and underrepresented minority status on faculty workload recommendations. *J. Women Minorities Sci. Eng.* 27 49–82. 10.1615/jwomenminorscieneng.2021034858

[B69] Kerber L. K. (1976). The republican mother: women and the enlightenment: an historical perspective. *Am. Q.* 28 187–205. 10.2307/2712349

[B70] Kriwy P. Gross C. Gottburgsen A. (2013). Look who’s talking: compositional effects of gender and status on verbal contributions at sociology conferences. *Gend. Work Organ.* 20 545–560. 10.1111/j.1468-0432.2012.00603.x

[B71] Kugler K. G. Reif J. A. Kaschner T. Brodbeck F. C. (2018). Gender differences in the initiation of negotiations: a meta-analysis. *Psychol. Bull.* 144 198–222. 10.1037/bul0000135 29239629

[B72] Laurin K. Kay A. C. Shepherd S. (2011). Self-stereotyping as a route to system justification. *Soc. Cogn.* 29 360–375. 10.1521/soco.2011.29.3.360

[B73] Leslie S. J. Cimpian A. Meyer M. Freeland E. (2015). Expectations of brilliance underlie gender distributions across academic disciplines. *Science* 347 262–265. 10.1126/science.1261375 25593183

[B74] Lundberg S. Stearns J. (2019). Women in economics: stalled progress. *J. Econ. Perspect.* 33 3–22. 10.1257/jep.33.1.3

[B75] Lynn F. B. Noonan M. C. Sauder M. Andersson M. A. (2019). A rare case of gender parity in academia. *Soc. Forces* 98 518–547. 10.1093/sf/soy126

[B76] Madera J. M. Hebl M. R. Martin R. C. (2009). Gender and letters of recommendation for academia: agentic and communal differences. *J. Appl. Psychol.* 94 1591–1599. 10.1037/a0016539 19916666

[B77] Magua W. Zhu X. Bhattacharya A. Filut A. Potvien A. Leatherberry R. (2017). Are female applicants disadvantaged in National Institutes of Health peer review? Combining algorithmic text mining and qualitative methods to detect evaluative differences in R01 reviewers’ critiques. *J. Women’s Health* 26 560–570. 10.1089/jwh.2016.6021 28281870 PMC5446598

[B78] Maliniak D. Powers R. Walter B. F. (2013). The gender citation gap in international relations. *Int. Organ.* 67 889–922. 10.1017/s0020818313000209

[B79] Mathews A. L. Andersen K. (2001). A gender gap in publishing? Women’s representation in edited political science books. *Polit. Sci. Polit.* 34 143–147. 10.1017/s1049096501000221

[B80] McAdam M. Harrison R. T. Leitch C. M. (2019). Stories from the field: women’s networking as gender capital in entrepreneurial ecosystems. *Small Bus. Econ.* 53 459–474. 10.1007/s11187-018-9995-6

[B81] McElhinny B. Hols M. Holtzkener J. Unger S. Hicks C. (2003). Gender, publication and citation in Sociolinguistics and linguistic anthropology: the construction of a scholarly canon. *Lang. Soc.* 32 299–328. 10.1017/s0047404503323012

[B82] Mershon C. Walsh W. (2016). Diversity in political science: why it matters and how to get it. *Polit. Groups Ident.* 4 462–466. 10.1080/21565503.2016.1170703

[B83] Miles M. L. Agger C. A. Roby R. S. Morton T. R. (2021). Who’s who: how “women of color” are (or are not) represented in STEM education research. *Sci. Educ.* 106 229–256. 10.1002/sce.21694

[B84] Mitchell S. M. Hesli V. L. (2013). Women don’t ask? Women don’t say no? Bargaining and service in the political science profession. *Polit. Sci. Polit.* 46 355–369. 10.1017/s1049096513000073

[B85] Mo C. H. (2015). The consequences of explicit and implicit gender attitudes and candidate quality in the calculations of voters. *Polit. Behav.* 37 357–395. 10.1007/s11109-014-9274-4

[B86] Morimoto S. A. (2022). The social science of institutional transformation: intersectional change in the academy. *Front. Psychol.* 7:824497. 10.3389/fsoc.2022.824497 PMC9049015 35495571

[B87] Morrow-Jones H. Box-Steffensmeier J. M. (2014). Implicit bias and why it matters to the field of political methodology. *Polit. Methodol.* 21 16–19.

[B88] Moss-Racusin C. A. Dovidio J. F. Brescoll V. L. Graham M. J. Handelsman J. (2012). Science faculty’s subtle gender biases favor male students. *Proc. Natl. Acad. Sci. U.S.A.* 109 16474–16479. 10.1073/pnas.1211286109 22988126 PMC3478626

[B89] National Center for Science and Engineering Statistics [NCSES] (2007). *National Science Foundation. ADVANCE: Increasing the Participation and Advancement of Women in Academic Science and Engineering Careers.* Alexandria, VA: National Science Foundation.

[B90] National Center for Science and Engineering Statistics [NCSES] (2017). *Survey of Doctorate Recipients, 2017.* Alexandria, VA: National Science Foundation.

[B91] National Center for Science and Engineering Statistics [NCSES] (2019). *Women, Minorities, and Persons with Disabilities in Science and Engineering: Data Tables.* Alexandria, VA: National Science Foundation.

[B92] National Center for Science and Engineering Statistics [NCSES] (2020). *Doctorate Recipients from U.S. Universities: 2019. NSF 21-308.* Alexandria, VA: National Science Foundation.

[B93] National Center for Science and Engineering Statistics [NCSES] (2021a). *Survey of Doctorate Recipients, 2019. NSF 21-320.* Alexandria, VA: National Science Foundation.

[B94] National Center for Science and Engineering Statistics [NCSES] (2021b). *Table 59. Median Annual Salaries of U.S. Residing Full-Time Employed Doctoral Scientists and Engineers in 4-Year Educational Institutions, by Field of Doctorate, Sex, and Faculty Rank: 2019. NSF 21-320.* Alexandria, VA: National Science Foundation.

[B95] National Science Foundation [NSF] (2017). *Women, Minorities, and Persons With Disabilities in Science and Engineering.* Alexandria, VA: National Science Foundation.

[B96] Niederle M. Segal C. Vesterlund L. (2013). How costly is diversity? Affirmative action in light of gender differences in competitiveness. *Manag. Sci.* 59 1–16. 10.1287/mnsc.1120.1602

[B97] Niederle M. Vesterlund L. (2007). Do women shy away from competition? Do men compete too much? *Q. J. Econ.* 122 1067–1101. 10.1162/qjec.122.3.1067

[B98] Nittrouer C. L. Hebl M. R. Ashburn-Nardo L. Trump-Steele R. C. Lane D. M. Valian V. (2018). Gender disparities in colloquium speakers at top universities. *Proc. Natl. Acad. Sci. U.S.A.* 115 104–108. 10.1073/pnas.1708414115 29255050 PMC5776791

[B99] O’Brien L. T. Major B. N. Gilbert P. N. (2012). Gender differences in entitlement: the role of system-justifying beliefs. *Basic Appl. Soc. Psychol.* 34 136–145. 10.1080/01973533.2012.655630

[B100] Odic D. Wojcik E. H. (2020). The publication gender gap in psychology. *Am. Psychol.* 75 92–103. 10.1037/amp0000480 31081649

[B101] Oliveira D. F. Ma Y. Woodruff T. K. Uzzi B. (2019). Comparison of National Institutes of Health grant amounts to first-time male and female principal investigators. *JAMA* 321 898–900. 10.1001/jama.2018.21944 30835300 PMC6439593

[B102] O’Meara K. A. Beise E. Culpepper D. Misra J. Jaeger A. (2020). Faculty work activity dashboards: a strategy to increase transparency. *Change* 52 34–42. 10.1080/00091383.2020.1745579

[B103] O’Meara K. A. Kuvaeva A. Nyunt G. Waugaman C. Jackson R. (2017). Asked more often: gender differences in faculty workload in research universities and the work interactions that shape them. *Am. Educ. Res. J.* 54 1154–1186. 10.3102/0002831217716767

[B104] Prentice D. A. Carranza E. (2002). What women and men should be, shouldn’t be, are allowed to be, and don’t have to be: the contents of prescriptive gender stereotypes. *Psychol. Women Q.* 26 269–281. 10.1111/1471-6402.t01-1-00066

[B105] Reuben E. Sapienza P. Zingales L. (2014). How stereotypes impair women’s careers in science. *Proc. Natl. Acad. Sci. U.S.A.* 111 4403–4408. 10.1073/pnas.1314788111 24616490 PMC3970474

[B106] Rissler L. J. Hale K. L. Joffe N. R. Caruso N. M. (2020). Gender differences in grant submissions across science and engineering fields at the NSF. *Bioscience* 70 814–820. 10.1093/biosci/biaa072 32973410 PMC7498325

[B107] Rudman L. A. Moss-Racusin C. A. Phelan J. E. Nauts S. (2012). Status incongruity and backlash effects: defending the gender hierarchy motivates prejudice against female leaders. *J. Exp. Soc. Psychol.* 48 165–179. 10.1016/j.jesp.2011.10.008

[B108] Sarsons H. (2015). *Gender Differences in Recognition for Group Work.* Cambridge, MA: Harvard University.

[B109] Schmader T. Whitehead J. Wysocki V. H. (2007). A linguistic comparison of letters of recommendation for male and female chemistry and biochemistry job applicants. *Sex Roles* 57 509–514. 10.1007/s11199-007-9291-4 18953419 PMC2572075

[B110] Smith J. L. Handley I. M. Zale A. V. Rushing S. Potvin M. A. (2015). Now hiring! Empirically testing a three-step intervention to increase faculty gender diversity in STEM. *BioScience* 65 1084–1087. 10.1093/biosci/biv138 26955075 PMC4777060

[B111] Smith J. L. Stoop C. Young M. Belou R. Held S. (2017). Grant-writing bootcamp: an intervention to enhance the research capacity of academic women in STEM. *BioScience* 67 638–645. 10.1093/biosci/bix050 29599541 PMC5862288

[B112] Smith N. (2014). *Economics is a Dismal Science for Women.* New York, NY: Bloomberg.

[B113] Steinpreis R. E. Anders K. A. Ritzke D. (1999). The impact of gender on the review of the curricula vitae of job applicants and tenure candidates: a national empirical study. *Sex Roles* 41 509–528. 10.1023/A:1018839203698

[B114] Streb M. J. Burrell B. Frederick B. Genovese M. A. (2008). Social desirability effects and support for a female American president. *Public Opin. Q.* 72 76–89. 10.1093/poq/nfm035

[B115] Teele D. Thelen K. (2017). Gender in the journals: publication patterns in political science. *Polit. Sci. Polit.* 50 433–447. 10.1017/S1049096516002985

[B116] United States Department of Labor (2021). *Equal Pay for Equal Work.* Available online at: https://www.dol.gov/agencies/oasam/centers-offices/civil-rights-center/internal/policies/equal-pay-for-equal-work (accessed October 9, 2021).

[B117] Vaid J. Geraci L. (2016). An examination of women’s professional visibility in cognitive psychology. *Feminism Psychol.* 26 292–319. 10.1177/0959353516641139

[B118] Van der Lee R. Ellemers N. (2015). Gender contributes to personal research funding success in The Netherlands. *Proc. Natl. Acad. Sci. U.S.A.* 112 12349–12353. 10.1073/pnas.1510159112 26392544 PMC4603485

[B119] Van Veelen R. Derks B. (2022). Equal representation does not mean equal opportunity: women academics perceive a thicker glass ceiling in social and behavioral fields than in the natural sciences and economics. *Front. Psychol.* 13:790211. 10.3389/fpsyg.2022.790211 35369222 PMC8966382

[B120] Weisshaar K. (2017). Publish and perish? An assessment of gender gaps in promotion to tenure in academia. *Soc. Forces* 96 529–560. 10.1093/sf/sox052

[B121] Welch K. Ruelas A. (2015). *The Role of Female Seminaries on the Road to Social Justice for Women.* Eugene: Wipf and Stock Publishers.

[B122] Winking J. Hopkins A. L. Yeoman M. Arcak C. (2019). M-AAA-nsplaining: gender bias in questions asked at the American Anthropological Association’s Annual Meetings. *PLoS One* 14:e0207691. 10.1371/journal.pone.0207691 30657759 PMC6338375

[B123] Women in Economics Initiative (2020). *Women in Economics Index.* Available online at: https://women-in-economics.com/wp-content/uploads/2020/10/WiE_Index_2020.pdf (accessed October 9, 2021).

[B124] Wong C. Y. Kirby T. A. Rink F. A. Ryan M. K. (2022). Intersectional invisibility in women’s diversity interventions. *Front. Psychol.* 10.3389/fpsyg.2022.791572 [Epub ahead of print]. PMC9176663 35693520

[B125] Wu A. H. (2020). Gender bias among professionals: an identity-based interpretation. *Rev. Econ. Stat.* 102 867–880. 10.1162/rest_a_00877

[B126] Zagorsky J. L. (2017). Divergent trends in U.S. maternity and paternity leave, 1994-2015. *Am. J. Public Health* 107 460–465. 10.2105/AJPH.2016.303607 28103064 PMC5296693

